# Extracellular vesicle microRNAs contribute to Notch signaling pathway in T-cell acute lymphoblastic leukemia

**DOI:** 10.1186/s12943-022-01698-3

**Published:** 2022-12-22

**Authors:** Tommaso Colangelo, Patrizio Panelli, Francesco Mazzarelli, Francesco Tamiro, Valentina Melocchi, Elisabetta De Santis, Roberto Cuttano, Orazio Palumbo, Giovanni Rossi, Fabrizio Bianchi, Vincenzo Giambra

**Affiliations:** 1 Unit of Cancer Biomarker, Fondazione IRCCS Casa Sollievo Della Sofferenza, Viale Padre Pio 7, 71013 San Giovanni Rotondo, FG Italy; 2 Unit of Hematopathology, Fondazione IRCCS Casa Sollievo Della Sofferenza, Viale Padre Pio 7, 71013 San Giovanni Rotondo, FG Italy; 3 Division of Medical Genetics, Fondazione IRCCS Casa Sollievo Della Sofferenza, 71013 San Giovanni Rotondo, FG Italy; 4 Department of Hematology and Stem Cell Transplant Unit, Fondazione IRCCS Casa Sollievo Della Sofferenza, 71013 San Giovanni Rotondo, FG Italy

**Keywords:** T-cell acute lymphoblastic leukemia, microRNAs, Exosomes, Extracellular vesicles, Gene expression

## Abstract

T-cell acute lymphoblastic leukemia (T-ALL) is an aggressive T-cell malignancy characterized by genotypically-defined and phenotypically divergent cell populations, governed by adaptive landscapes. Clonal expansions are associated to genetic and epigenetic events, and modulation of external stimuli that affect the hierarchical structure of subclones and support the dynamics of leukemic subsets. Recently, small extracellular vesicles (sEV) such as exosomes were also shown to play a role in leukemia. Here, by coupling miRNome, bulk and single cell transcriptome profiling, we found that T-ALL-secreted sEV contain NOTCH1-dependent microRNAs (EV-miRs), which control oncogenic pathways acting as autocrine stimuli and ultimately promoting the expansion/survival of highly proliferative cell subsets of human T-cell leukemias. Of interest, we found that NOTCH1-dependent EV-miRs mostly comprised members of miR-17-92a cluster and paralogues, which rescued in vitro the proliferation of T-ALL cells blocked by γ-secretase inhibitors (GSI) an regulated a network of genes characterizing patients with relapsed/refractory early T-cell progenitor (ETP) ALLs. All these findings suggest that NOTCH1 dependent EV-miRs may sustain the growth/survival of immunophenotypically defined cell populations, altering the cell heterogeneity and the dynamics of T-cell leukemias in response to conventional therapies.

**Supplementary Information:**

The online version contains supplementary material available at 10.1186/s12943-022-01698-3.

## Background

Acute lymphoblastic leukemia or ALL, is an aggressive malignancy of immature lymphocytes with about 15–20% of cases of T lineage (T-ALL). It is the most common type of cancer in children, but also affects adults with incidences of ~ 30 new cases per 1,000,000 per year [1]. Pediatric T-ALL is largely curable with intensive chemotherapy, but there are significant side effects and ~ 20% of patients suffer relapse. In contrast, adult T-ALL is characterized by a 5-year overall survival of ~ 40% [2].

T-ALL is the result of a malignant alteration of hematopoietic progenitors during T-cell development. A relevant oncogenic pathway involved in T-cell transformation is the NOTCH1 signaling pathway with over 50% of human T-ALL carrying activating mutations of NOTCH1 gene [3, 4].

Recently, small extracellular vesicles (sEV) such as exosomes were reported to contribute to leukemic progression [5, 6]. sEV were shown to reprogram the bone-marrow microenvironment [7], dampen anti-leukemia immune response [8] and promote drug resistance [9]. sEV exert such molecular and cellular functions by transferring molecular information from cancer cells to proximal and/or to distant body districts, including pro-metastatic niche [10]. Importantly, in T-ALL a miRNA-tumor suppressor gene network drives the malignant transformation of T-cell progenitors [11, 12] and cooperates with NOTCH1-driven T-ALL [13–17]. However, the precise role of sEV and miRNA cargo in NOTCH1-driven T-ALL remains elusive. Here, we tackle this issue and present new evidences supporting a central role for EV-miRs in the progression of NOTCH1-driven T-ALL.

## Results and discussion

**Fig. 1:**
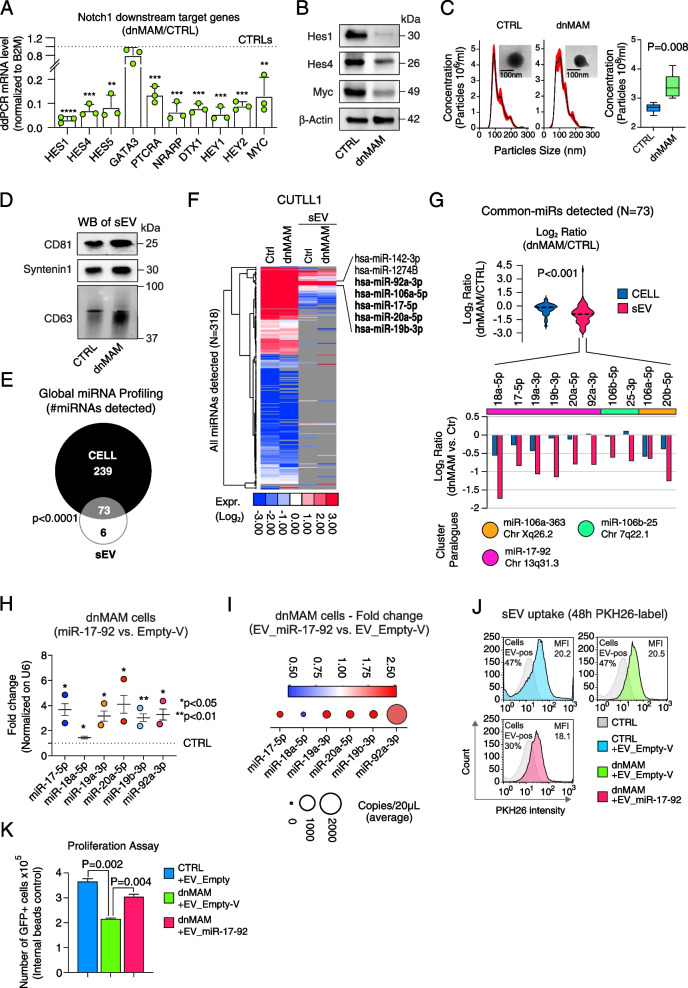
EV-miRNAs characterization and function in T-ALL model. **A** ddPCR analysis of validated NOTCH1 target genes mRNA expression in CUTLL1-dnMAM vs. CUTLL1-CTRL cells. Y-axes, mRNA levels of NOTCH1 target genes normalized to B2M expression. X-axes, gene symbols. Significance analysis was performed by one-sample t-test. **B** Immunoblot analysis of HES1, HES4 and c-MYC proteins in CUTLL1-CTRL and CUTLL1-dnMAM cells. **C** Nanoparticle-tracking-analysis of the size distribution and concentration of sEV released by CUTLL1-CTRL and CUTLL1-dnMAM cells. Inset plots, Transmission Electron Microscope (TEM) images showing particles in sEV samples from CUTLL1-CTRL and CUTLL1-dnMAM cells. Scale bar = 100 nm. On the right, box plots of differential concentration of sEV in CUTLL1-CTRL and CUTLL1-dnMAM cells. Significance analysis was performed by Student t-test. **D** Immunoblot analysis of sEV markers (CD81, Syntetin1 and CD63) in CUTLL1-CTRL and CUTLL1-dnMAM cells. **E** Venn diagram of EV-miRNAs detected in CUTLL1-CTRL and CUTLL1-dnMAM cells (CELL) or in their released small extracellular-vesicles (sEV). Significance analysis was performed by Fisher’s exact test. **F** Hierarchical clustering analysis of miRNAs detected (*N* = 318) in CUTLL1-CTRL (Ctrl) and CUTLL1-dnMAM (dnM) cells (CELL) and/or in their released small extracellular-vesicles (sEV). On the right, most abundant miRNAs in sEV were also indicated; in bold, members of the miR-17–92 cluster. **G** On top, violin plots of differential expression (dnMAM vs. CTRL) of the 73 commonly detected miRNAs in CUTTL1 cells and in sEV. Bottom, bar plots of differential expression (dnMAM vs. CTRL) of the miR-17–92 cluster and paralogues. Colors are as per the legend. Significance analysis was performed by Mann–Whitney U-test. **H** qRT-PCR analysis of miR-17–92 cluster overexpressing CUTLL1-dnMAM cells vs. control (Empty-V) CUTLL1-dnMAM cells. Significance analysis was performed by one-sample t-test. **I** ddPCR analysis of miR-17–92 cluster in sEV purified from miR-17–92 cluster overexpressing vs. control (Empty-V) CUTLL1-dnMAM cells. Bubble size represents the average expression of miRNAs (copies/20µL). Colours are as per the legend. **J** Flow cytometry analysis of CUTTL1 (CTRL) and CUTTL1-dnMAM cells (dnMAM) conditioned with PKH26-labelled miR-17–92-enriched sEV (EV_miR-17–92) or PKH26-labelled Empty-Vector sEV (EV_Empty-V) derived from miR-17–92 overexpressing CUTTL1 cells or from CUTLL1 cells transfected with an empty vector, respectively. MFI, mean fluorescence intensity. Percentages of cells which internalized exogenous PKH26-sEV (Cells EV-pos) are also shown. **K** Viability of CUTTL1 (CTRL) and CUTTL1-dnMAM cells (dnMAM). Briefly, transduced GFP positive cells were FACS sorted and in vitro grown together with miR-17–92-enriched sEV (EV_miR-17–92) or sEV (EV_Empty-V) cultured for two days. GFP + alive cells were measured by flow cytometry for DAPI (4′,6-diamidino-2-phenylindole) exclusion and counted by relating the cell numbers to internal fluorescent bead events (see also methods). The graph reports the result of two independent experiments. Significance analysis was performed by Student’s t-test

**Fig. 2:**
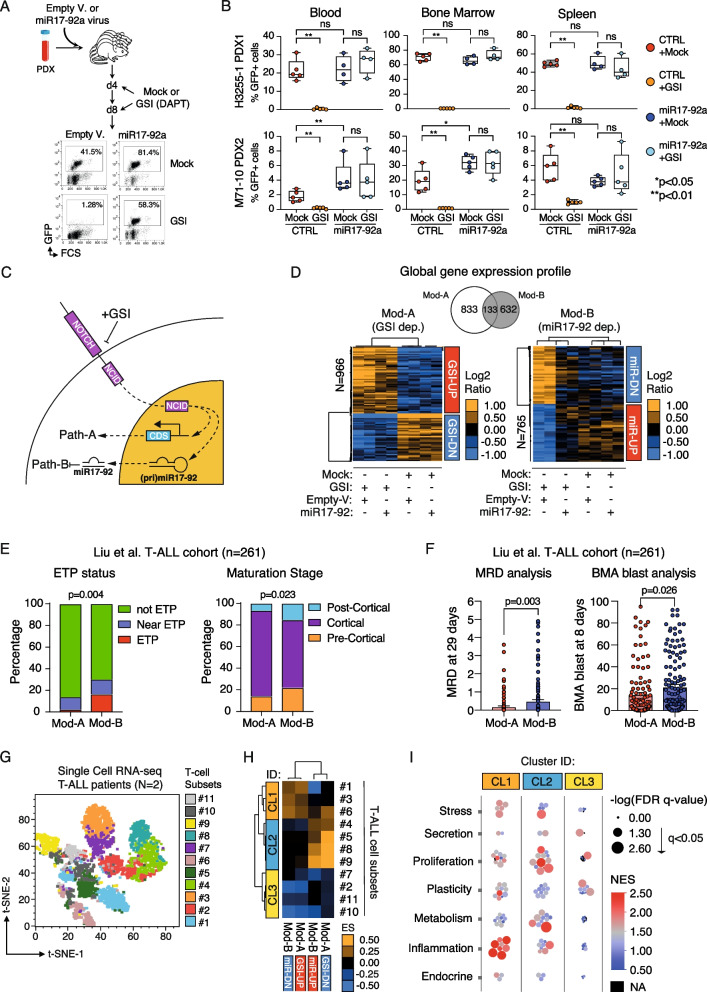
Insights in biological and molecular functions of miR-17–92 cluster in T-ALL. **A** Schematic diagram of the approach used for PDX T-ALLs transduced with lentiviral constructs encoding miR-17–92 cluster or empty as a control (Empty V.) and cultured for 3 days on MS5-DL1 feeders. Transduced (GFP +) cells were then sorted with FACS and transplanted into immunodeficient NSG recipient mice, which were subsequently treated with DAPT γ-secretase inhibitor (1 mg/mouse) or dimethyl sulfoxide (DMSO), both delivered by intraperitoneal injection at days 4 and 8 post-transplant. **B** Flow cytometry analysis of GFP + CD45 + alive cells in peripheral blood, bone marrow or spleen from transplanted recipient mice treated as describe in (A). Y-axes, percentage of GFP + cells. X-axes, experimental conditions. Significance analysis was performed by Mann–Whitney U test. **C** Graphical representation of canonical (Mod-A) and of miR-17–92 modulated NOTCH1-signalling pathway (Mod-B). **D** Hierarchical clustering analysis of Mod-A and Mod-B gene expression profile in the various experimental conditions (i.e., ± GSI; miR-17–92 OE or empty vector). Main clusters of genes are also indicated as GSI-UP/GSI-DOWN (Mod-A) or miR-UP/miR-DOWN (Mod-B). Colours are as per the legend. On top, Venn diagram showing Mod-A/B number of genes and relative overlapping. **E** Percentage distribution of ‘ETP status’ and ‘Maturation stage’ of T-ALLs in the Liu et al. cohort (*n* = 261) stratified according to ssGSEA using Mod-A (*n* = 123) and Mod-B (*n* = 138) gene sets. ETP, Early T-cell Precursor. *P*-values were computed by chi-square test. **F** Box-plots show the levels of MRD (at 29 days) and BMA of blasts (at 8 days) in T-ALLs the Liu et al. cohort (*n* = 261) stratified as in (D). MRD, Minimal Residual Disease. BMA, Bone Marrow Aspirates. **G** t-SNE plot of scRNAseq data on cell subsets of PDX from T-ALL patients. Colours are as per the legend. **H** Hierarchical clustering analysis of Enrichment Scores from GSEA using the Mod-A (GSI-UP/DOWN) and Mod-B (miR-UP/miR-DOWN) gene sets in the T-ALL cell subsets profiled by scRNAseq as in (**G**). **I** Distributions of enrichment of biological functions which were identified by GSEA using scRNA-expression profiles, in clusters of T-ALL cell subsets as in (**H**). The higher the size of bubbles the more significant is the enrichment of a particular biofunction. Colors of bubbles indicate the magnitude of normalized enrichment scores (NES) and are as per the legend

The molecular characteristics of sEV in T-ALL were initially explored in CUTLL1 cell line, a well-characterized human T-cell lymphoma cell line derived from a pleural effusion in a pediatric patient with T-ALL at relapse, with aberrant NOTCH1 activation and strongly sensitive to γ-secretase inhibitors [18]. CUTLL1 cells were lentivirally transduced to constitutively express a dominant-negative form of Mastermind-like protein 1 (dnMAM) to shutdown NOTCH1 signalling, or an empty vector as a control. Indeed, several NOTCH1 target genes were strongly reduced under dnMAM condition (Fig. 1A, B; Fig. S1A). Next, we analyzed size distribution, morphology, quantities of sEV released from CUTLL1-CTRL and CUTLL1-dnMAM cells by nanoparticle-tracking (NTA), TEM, and WB analyses using sEV markers (Fig. 1C-D; Fig. S1B-C). Overall, the prevalent size of sEV matched with expected exosome size distribution (i.e., ~ 30-150 nm; Fig. 1C) and sEV concentration was significantly increased in dnMAM cells (Fig. 1C) in line with previous results showing that a deranged NOTCH signaling in T cells induces a dramatic increase in exosomes release [19]. We detected a total of 318 miRNAs (Fig. 1E; Table S1) by whole-miRNA expression profiling of CUTTL1 cells of which 73 also detected in sEV (i.e., Common-miRs; Fig. 1E; Table S1). Yet, hierarchical clustering analysis showed a set of highly abundant ‘EV-miRs’ comprising members of miR-17-92a cluster and paralogues (Fig. 1F), which we found to be characterized by overrepresented EXOmotifs (Figure S1D), i.e. sorting sequences that determine miRNAs upload into sEV [20, 21]. In line with previous reports, miR-19b is highly expressed in T-ALL cells and is targeted by the t(13;14)(q32;q11) translocation in T-ALL. Likewise, other members of miR-17-92a clusters i.e., miR-20a and miR-92a, were found highly expressed in T-ALL and together with miR-19b were shown being capable of promoting T-ALL [11]. Expression profiling analysis of the Common-miRs in T-ALL cells and in sEV (Fig. 1E), revealed a significant and specific decreased expression of miR-17–92 cluster and paralogues upon NOTCH1 signalling inactivation (i.e., dnMAM vs. CTRL; Fig. 1G) both intracellularly and in sEV (Fig. 1G), which suggests NOTCH1 signalling modulates EV-miRs quantities in sEV. As matter of fact, previous studies showed the existence of a tight interplay between c-MYC and miR-17–92 cluster expression [22] and that c-MYC is an important direct target of Notch-1 in T-ALL [23]. Consistently, we found that miR-17–92 cluster expression is rescued in dnMAM condition upon forced expression of c-MYC (Fig. S1E-G). Next, to investigate the function of these NOTCH1-dependent EV-miRs, we produced PKH26-labelled sEV enriched in miR-17–92 (aka EV_miR-17–92) by overexpressing miR-17–92 cluster in dnMAM cells which yielded sEV enriched in miR-17–92 cluster (Fig. 1H-I; see methods). Internalization of EV_miR-17–92 in dnMAM cells (Fig. 1J) significantly increased the proliferation rate to a comparable level to NOTCH1-proficient CUTLL1 CTRL cells (Fig. 1K). We then treated CUTLL1-wt cells with γ-secretase inhibitor (GSI; see supplemental methods) and observed, as expected, a strong impairment of cell viability (Fig. S2A). Contrariwise, EV_miR-17–92 induced expansion of CUTLL1-wt cells (*p* < 0.01; Student's T-test; Fig. 2A) and, importantly, were able to rescue the GSI-induced phenotype in T-ALL cells (Fig. 2A). Similar results were obtained by using cells from two independent clones of T-ALL patient-derived xenografts (PDX) (Fig. S2B). Of note, using a known NOTCH1-dependent miRNA i.e. the miR-223-3p (Table S1; [24, 25]) we obtained comparable results in vitro (Fig. S2C-E). Finally, leukemia cells of M71 and H3255 PDX lines were transduced with lentiviruses encoding miR-17–92 cluster or empty vector (EV) as control and subsequently transplanted into immunocompromised (NSG) mice. In line with all previous results, we observed that miR-17–92 transduced human cells indeed rescued the GSI-induced phenotype in T-ALL PDXs (Fig. 2A-B).

Taken together, such results showed, for the first time, the ability of sEV_miR-17–92 to propagate molecular information among T-ALL cells which was able to restore, at least in part, a defective NOTCH1 signalling pathway.

Lastly, we dissected the molecular function of miR-17–92 cluster in the realm of NOTCH1-driven T-ALL. We reasoned that NOTCH1 signalling can be generalized in two main routes: Path-A) the ‘canonical’ transcriptional output of NOTCH1 intracellular domain (NCID) (Fig. 2C); Path-B) the transcriptional output controlled by NOTCH1 through miR-17–92 (Fig. 2C). High-throughput gene expression profiling of CUTLL1 cells ± miR-17–92, and GSI/mock treated (Fig. 2D; see methods) followed by quantitative trait analysis (see methods) identified two transcriptional gene modules, i.e. Mod-A (*N* = 966 genes) and Mod-B (*N* = 765 genes), which differ in terms of transcriptional regulation and are both dependent to GSI treatment yet indifferent to rescued miR-17–92 expression (i.e., Mod-A; Fig. 2D; see methods), or reverted (i.e., Mod-B Fig. 2D) (Table S2). Such results confirmed our hypothesis of a bipartite NOTCH1 signalling transcriptional output (Fig. 2C). Notably, MSigDB analysis of Mod-B gene sets revealed a strong and significant enrichment (FDR q-value < 0.0001) of predicted miR-17–92-targeted transcripts (Table S3; see methods) further confirming the regulatory function of the miR-17–92 cluster in Mod-B. Furthermore, IPA software (see methods) revealed that Mod-A comprised canonical NOTCH-signalling genes (e.g., NOTCH2-4, c-MYC, CTNNB1, GATA1-3, etc.) (Fig. S3A) while Mod-B was enriched in gene involved in proliferation (CDKN2A, CCNE1, E2F3, E2F6, RBL1), stemness (FOXM1, TCF4) and cancer (ETS1, RELA, NFE2L2) (Fig. S3B). Intriguingly, when we used Mod-A and Mod-B gene sets to stratify an external cohort of human T-ALL (i.e., the Liu et al. cohort, *N* = 261; Table S4; [26]), we observed that Mod-B gene set hallmarks T-ALLs particularly enriched in the Early T-cell precursor (ETP) and pre-/post-cortical subtypes, with a higher post-therapeutic minimal residual disease (MRD), and blast count in the bone marrow, that are all characteristics of an adverse outcome [27–29](Fig. 2E-F; Table S5).

Next, we performed single-cell RNA sequencing of primary cells, derived from T-ALL patients (*N* = 2), without any expansion in vivo into immunocompromised mice. Using the Phenograph algorithm [30], we identified several distinct cell subsets (*n* = 11) (Fig. 2G). Gene set enrichment analysis (GSEA) using Mod-A and -B and hierarchical clustering analysis revealed three main clusters grouping T-ALL cell subsets which shared similar pattern of enrichment scores (ES) (Fig. 2H). In particular, CL2 contains cell subsets contributed by both two patients (Fig. S2F-G) with coherent expression trend of both Mod-A and -B as defined in Fig. 2D, which is a hallmark of activity of NOTCH1 signalling pathway. Indeed, GSEA using Hallmark gene sets (see methods) confirmed that these CL2-cell subsets were significantly characterized by mechanisms involved in proliferation and metabolism (Fig. 2I), which further show how both canonical’ NCID signalling (Mod-A) and NOTCH1 miR-17–92 mediated signalling (Mod-B) can contribute to Notch1-related phenotypes and coexist in the same T-ALL cell subsets.

Our findings shed new light on composite interactions between sEV-miRs, Notch signalling and cellular plasticity that characterize the tumor heterogeneity of T-ALL and promote relapsed/refractory cell subsets of T-cell leukemias. In this scenario, further investigations are needed to explore such mechanisms in T-ALL with the final intent of offering more efficient therapies targeting diverse oncogenic states and microenvironments that support aggressive tumor cells.

## Materials and methods

For extensive details on all methodologies see online Supplemental Material and Methods.

### Human samples

The institutional ethical committees approved this study (registration number: N91/CE), and informed consent was obtained from all patients enrolled.

### Profiling by TaqMan Human MicroRNA Arrays

Expression levels of 754 miRNAs were quantified using the TaqMan Human MicroRNA Array A + B Card Set v3.0 (Applied Biosystems, Foster City, CA).

### Genome-wide expression profiling

Gene expression profiling was performed using the GeneChip® Human Clarion S Array (Thermo Fisher Scientific) including more than 210,000 distinct probes representative of > 20,000 well-annotated genes (hg19; Genome Reference Consortium Human Build 37 (GRCh37)).

### Single cell RNA-sequencing (scRNA-Seq)

Whole transcriptome analysis at single cell level was performed on FACS-sorted primary T-ALL cells using the BD Rhapsody Single-Cell Analysis System (BD, Biosciences).

### Data set availability

The normalized (U6) data for miRNA can be found in Table S1 while mRNA expression data can be accessible at NCBI GEO (GSE193482) and SRA (PRJNA784728 for scRNA-Seq data). 

## Supplementary Information

**Additional file 1.**

**Additional file 2.**

**Additional file 3.**

**Additional file 4.** Supplemental materials and methods.

**Additional file 5: Table S1.** miRNA normalized (U6) expression data. **Table S2.** Mod-A and Mod-B gene sets. **Table S3.** MSigDB analysis of Mod-B genes. **Table S4.** Liu et al. T-ALL cohort stratified according to ssGSEA analysis performed with Mod-A and Mod-B gene sets (N=261 patients; see supplemental materials and methods). **Table S5.** Clinicopathologic characteristics of Liu et al. T-ALL cohort stratified according to ssGSEA analysis performed with Mod-A and Mod-B gene sets (N=261 patients; see supplemental materials and methods).

## Data Availability

The normalized (U6) data for miRNA can be found in Table S1 while mRNA expression data can be accessible at NCBI GEO (GSE193482; reviewer token: gjubkewovfsvdyr) and SRA (PRJNA784728 for scRNA-Seq data).

## Abbreviations

T-ALL: T-cell acute lymphoblastic leukemia
sEV: Small extracellular vesicles
EV-miRs: SEV contain NOTCH1-dependent microRNAs
GSI: γ-Secretase inhibitors
ETP: Early T-cell progenitor
dnMAM: Dominant-negative form of Mastermind-like protein 1
NTA: Nanoparticle-tracking analysis
PDX: Patient-derived xenografts
NCDI: NOTCH1 intracellular domain
MRD: Minimal residual disease
GSEA: Gene set enrichment analysis
IPA: Ingenuity Pathway Analysis
ddPCR: Droplet Digital PCR
CTRL: Control
NES: Normalized enrichment scores
