# Supplementary figures and images for: Extracellular vesicle microRNAs contribute to Notch signaling pathway in T-cell acute lymphoblastic leukemia
Source: Mol Cancer. 2022 Dec 22;21:226. doi: 10.1186/s12943-022-01698-3 (PMC9773489; doi:10.1186/s12943-022-01698-3)

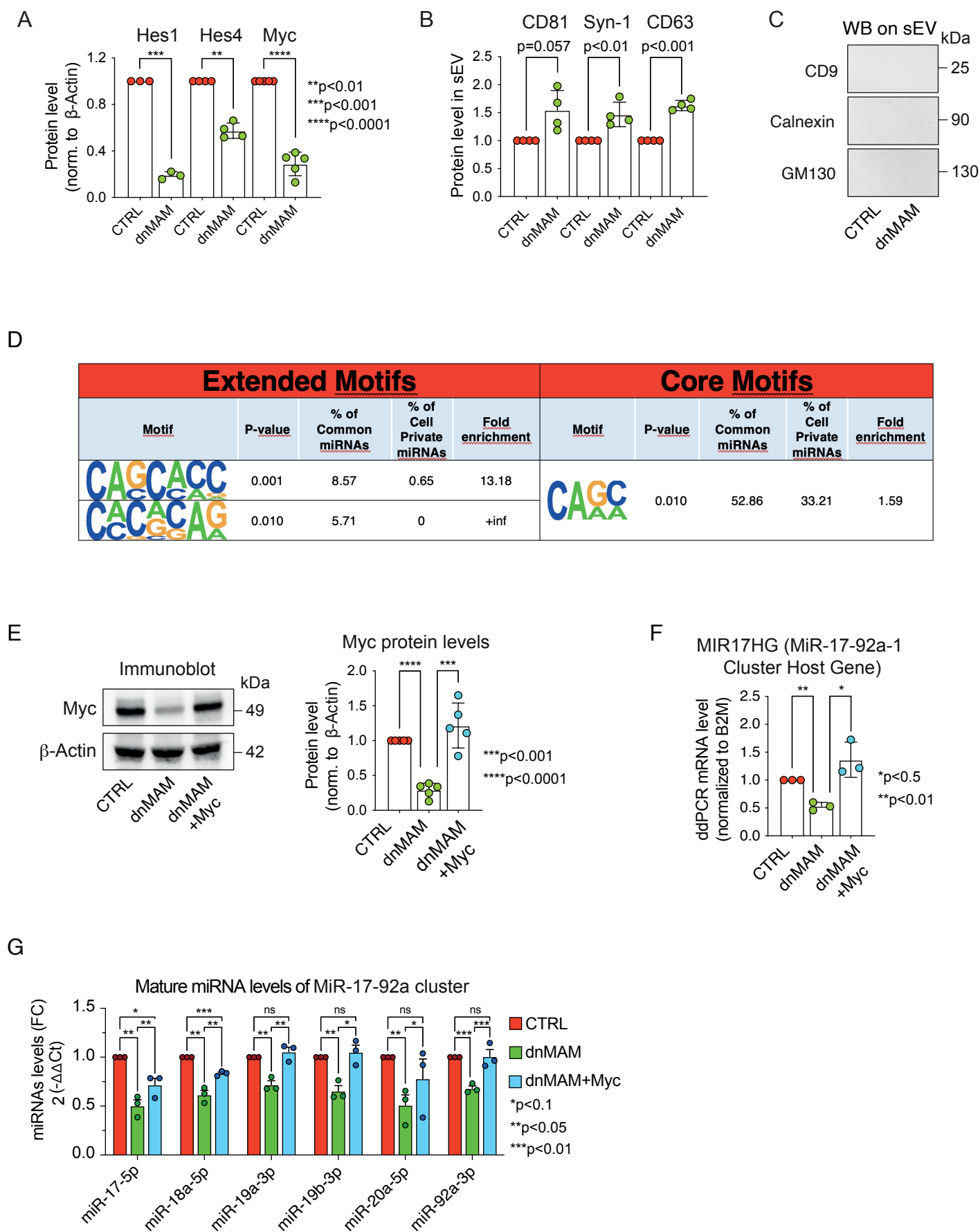

Supplement: Supplementary file 1 — Additional file 1. [file 12943_2022_1698_MOESM1_ESM.pdf]

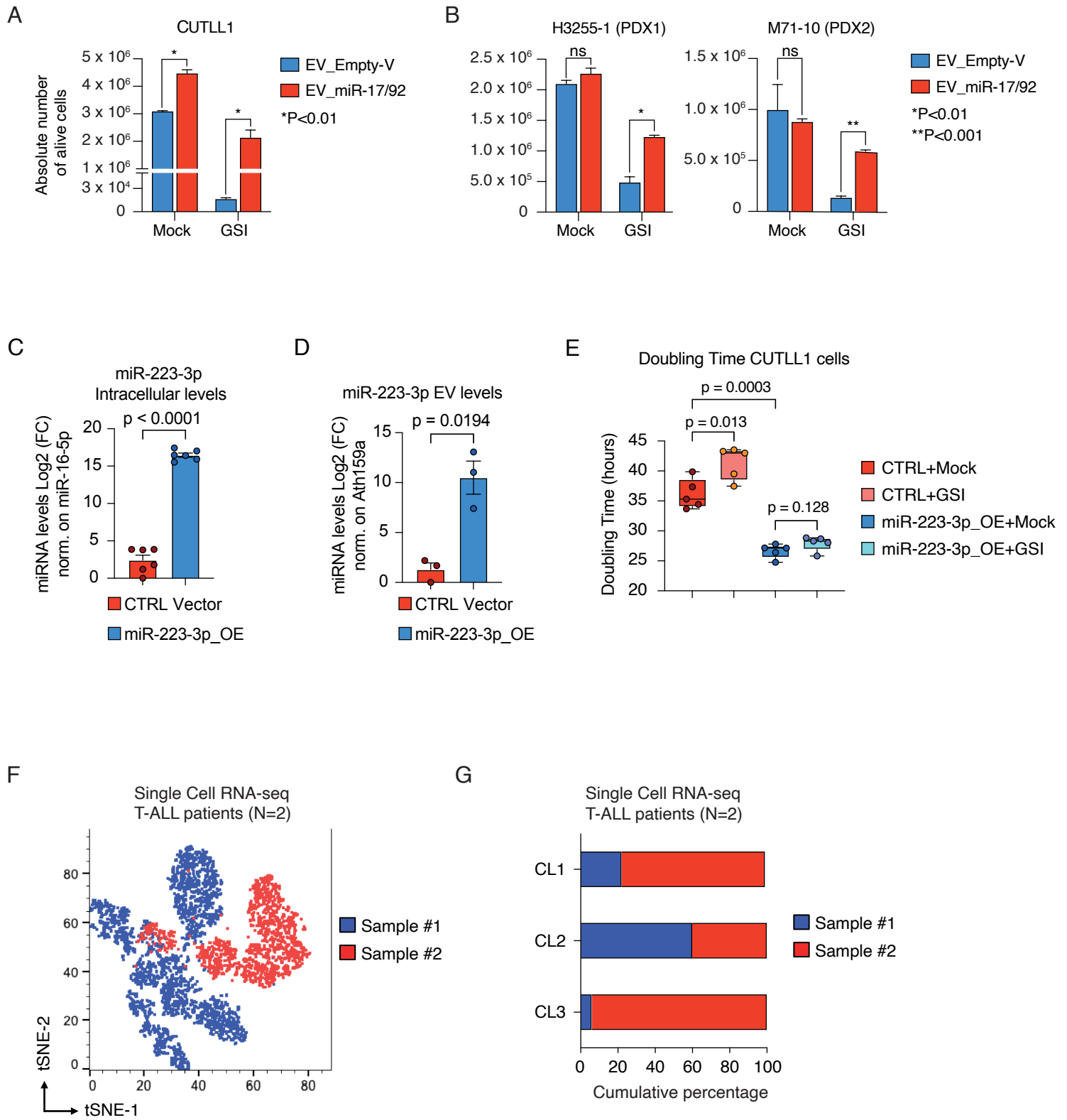

Supplement: Supplementary file 2 — Additional file 2. [file 12943_2022_1698_MOESM2_ESM.pdf]

A

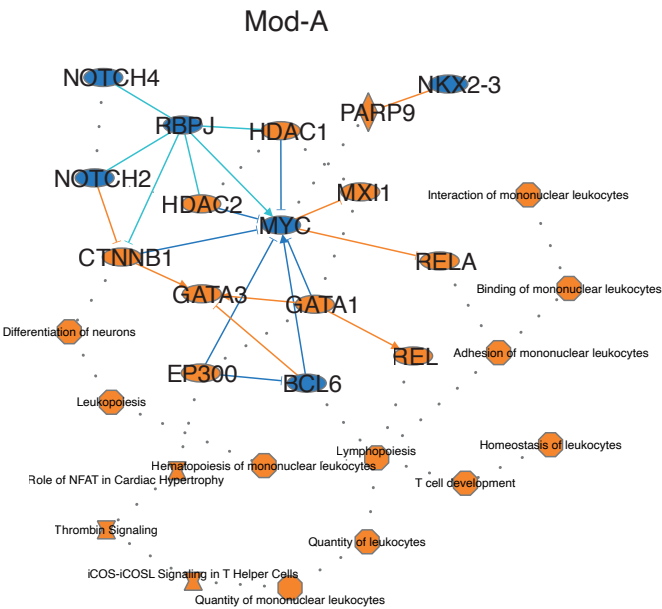

B

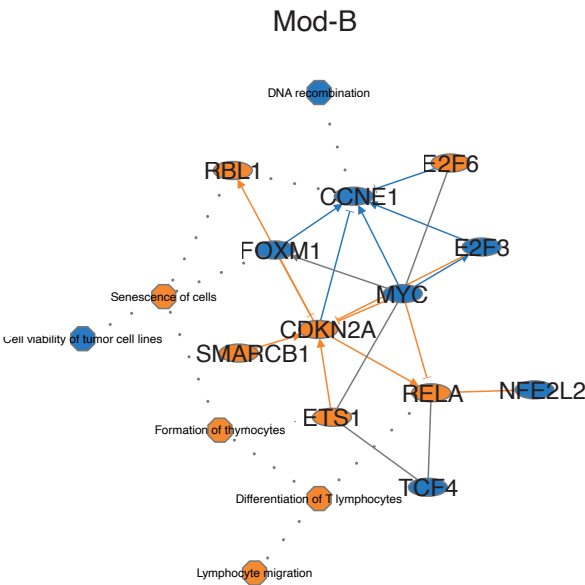

Supplement: Supplementary file 3 — Additional file 3. [file 12943_2022_1698_MOESM3_ESM.pdf]
